# Laboratory features of tumour lysis syndrome following daratumumab monotherapy in relapsed/refractory multiple myeloma

**DOI:** 10.1002/jha2.34

**Published:** 2020-07-04

**Authors:** Louisa Shackleton, James Fay, Elizabeth Smyth, Philip Murphy, Siobhan Glavey, John Quinn

**Affiliations:** ^1^ Department of Haematology Beaumont Hospital Dublin Ireland

Dear Editor,

We read with great interest the recent report by Yavorkovsky et al (BJH 2020) where the authors document three separate cases of tumour lysis syndrome (TLS) in the setting of multiple myeloma (MM) therapy [1]. The authors postulate that an abrupt increase in serum‐free light chain (SFLC) concentration in the setting of abrupt MM cell death may contribute significantly to the acute kidney injury seen in TLS [1]. TLS is rarely seen in multiple myeloma (MM) but has been reported following treatment with the proteasome inhibitors (PI) bortezomib and carfilzomib [2, 3]. However, less is known about the risk of TLS with other anti‐myeloma agents including immunomodulatory drugs (iMIDS) and monoclonal antibodies. Daratumumab is an anti‐IgG1 monoclonal antibody that has significant anti‐myeloma activity, both as a single agent and in combination with other anti‐myeloma agents in the setting of both de novo and relapsed disease [4, 5]. To the best of our knowledge, TLS has not been reported in the setting of daratumumab monotherapy in MM and therefore here we describe a case where laboratory evidence of TLS was observed after treatment with single‐agent daratumumab.

An 83‐year‐old lady with relapsed/refractory light‐chain MM was admitted to hospital with progressive disease. Her medical history included congestive cardiac failure, asthma, melanoma and pulmonary embolism. She was diagnosed with MM 2 years previously and at diagnosis had renal impairment requiring dialysis (serum creatinine was 402 μmol/L and calcium 2.42 mmol/L). Serum protein electrophoresis (SPEP) showed a small IgD kappa paraprotein and SFLC showed kappa > 1800 mg/L with lambda 5.4 mg/L (ratio > 337). Bone marrow trephine biopsy showed 70% kappa‐restricted plasma cells and skeletal survey showed widespread lytic bone disease. She commenced bortezomib and dexamethasone (VD) therapy and responded well with best SFLC ratio being 2.12 (kappa 34.4 mg/L) after 3 months of therapy. Indeed, she had become independent of dialysis following 2.5 months of treatment with subsequent normalisation of serum creatinine. The patient completed eight cycles of VD but relapsed 3 months later and commenced lenalidomide plus dexamethasone (RD). Five months later, her MM again progressed and RD was adjusted to lenalidomide plus low‐dose alternate day cyclophosphamide 50 mg OD PO and prednisolone 20 mg OD PO. The SFLCr remained stable for a further 15 months before it again rose to 2640 (kappa 4828 mg/L). The patient was then admitted to hospital to commence single‐agent daratumumab and her admission laboratory parameters are outlined in Table 1. She was pre‐medicated with intravenous (IV) fluids, oral allopurinol 100 mg OD PO and IV methylprednisolone. The patient commenced daratumumab 16 mg/kg at a rate of 50 mL per hour but 90 minutes into the infusion with approximately 10% of dose administered, she developed an infusion‐related reaction (IRR), becoming tachycardic, hypertensive and dyspnoeic, and requiring 100% oxygen to maintain SpO_2_ 95%. The infusion was discontinued and she was treated with IV hydrocortisone, IV chlorphenamine and nebulised ipratropium bromide. Although the symptoms quickly resolved, the infusion was not recommenced. The laboratory abnormalities associated with this reaction are outlined below and meet the criteria for laboratory TLS (25% increase from baseline of uric acid and phosphate). The patient was treated for TLS with 2 days of IV rasburicase 0.2 mg/kg and IV hydration until laboratory parameters normalised. A repeat 10% dose of daratumumab 10 days later was well tolerated without any evidence of TLS (Table 1) having been pre‐treated with dexamethasone and an oral leukotriene antagonist. She was subsequently treated with full‐dose daratumumab 1 week later, following IV hydration for prevention of TLS and pre‐treatment with rasburicase. Although no clinical adverse reaction was observed, her blood results did again show evidence of laboratory TLS as outlined below (25% increase from baseline of uric acid, potassium and phosphate). Unfortunately, our patient subsequently developed a hospital‐acquired pneumonia (HAP) and clinically deteriorated. She made a decision to forego any additional anti‐myeloma therapy and received palliative care, meaning that any follow‐up assessment of SFLCr and other disease parameters was not undertaken.

**TABLE 1:** Laboratory results pre‐ and post‐daratumumab infusions

Laboratory parameters	Pre‐DARA #1	Post‐DARA #1	Pre‐DARA #2	Post‐DARA #2	Pre‐DARA #3	Post‐DARA #3
Potassium 3.5‐5.3 mmol/L	4.6	4.6	3.2	3.1	3.4	4.9
Urea 2.8‐8.1 mmo/L	16.9	20.7	12.8	10.7	6.0	8.9
Creatinine 45‐84 μmol/L	198	198	127	112	135	145
Calcium 2.15‐2.5 mmol/L	2.63	2.53	2.13	2.4	2.62	2.57
Phosphate 0.81‐1.45 mmol/L	1.75	2.52	0.93	0.44	1.14	2.32
Urate 143‐340 μmol/L	618	906	N/A	N/A	459	577
LDH 208‐378 units/L	N/A	5577	568	472	Haemolysed	4560

Abbreviations: DARA, daratumumab; N/A, not available.

MM is typically a slowly proliferating tumour and has a low incidence of TLS, estimated to occur in approximately 1% of cases of MM [6] The Cairo‐Bishop TLS criteria denote laboratory TLS as being defined by specific electrolyte abnormalities due to loss of intracellular contents—hyperkalemia, hyperuricaemia, hyperphosphatemia, hypocalcaemia and deranged renal function, while clinical TLS requires additional clinical manifestations due to these electrolyte abnormalities, for example, AKI, cardiac arrhythmia or seizures [7]. Although our patient did not develop clinical complications, she did meet the criteria for a diagnosis of laboratory TLS. There is sparse literature documenting the frequency of TLS in MM, however some studies suggest a higher frequency of TLS with PI‐based therapy. For example, a Japanese retrospective study of 64 patients found that out of a total of 124 chemotherapy regimens, TLS occurred in 13 out of the 124 courses (10.5%) [2] and also showed that the incidences of TLS were 17.5% and 3.2% for the bortezomib and non‐bortezomib‐based regimens, respectively, while no TLS occurred in patients treated with immunomodulatory drug (IMiD) containing regimens [2].

Reports of TLS associated with single‐agent monoclonal antibody therapy are very rare in haematological cancer. For example, the SIRIUS study reported on the outcomes of 106 MM patients with relapsed/refractory MM treated with single‐agent daratumumab and no cases of TLS or hyperphosphatemia were reported [4]. TLS was reported following treatment with elotuzumab, however the picture in this case was complicated by pre‐treatment with lenalidomide and concomitant medications [8]. In a case of chronic lymphocytic leukaemia (CLL), a single test dose of obinutuzumab led to TLS and rapid tumour reduction [9].

In summary, we present the case of an 83‐year‐old lady who developed laboratory TLS following treatment with single‐agent daratumumab. In keeping with the cases described by Yavorkovsky et al, our patient had a high burden of light chain secreting disease. We wish to highlight a very rare but potential complication of monoclonal antibody treatment in MM patients with large tumour burden and/or those with rapidly proliferating tumours. As daratumumab and other monoclonal antibodies become more widely used in both de novo and relapsed/refractory MM (and in other cancers); it is important that the potential for TLS is borne in mind and prophylactic measures are considered.

## AUTHOR CONTRIBUTIONS

Louisa Shackleton, James Fay, and Elizabeth Smyth wrote the paper. John Quinn, Siobhan Glavey, and Philip Murphy reviewed and edited the paper.
